# ‘Mr Cummings clearly does not understand the science of genetics and should maybe go back to school on the subject’: an exploratory content analysis of the online comments beneath a controversial news story

**DOI:** 10.1186/s40504-016-0044-4

**Published:** 2016-11-03

**Authors:** Madeline Crosswaite, Kathryn Asbury

**Affiliations:** Department of Education, Derwent College, University of York, York, YO10 5DD UK

## Abstract

An article published in the UK Guardian on 11/10/2013 with the headline ‘Genetics outweighs teaching, Gove advisor tells his boss’ reported a leaked document written by special advisor Dominic Cummings to the then UK Secretary of State for Education, Michael Gove. The article generated 3008 on-line reader comments from the public. These reader comments offer a naturalistic opportunity to understand public opinion regarding Cummings’ controversial suggestions and ideas. We conducted a content analysis of *n* = 800 reader comments, coding them on the basis of level of agreement with the ideas and opinions expressed in the article. Of all aspects of education mentioned, Cummings’ reported views on genetics were commented upon most frequently and were subject to the most opposition from commenters, but also the most support. Findings offer some insight into the challenges involved in conducting public discourse about the relevance of genes in education. We discuss the accuracy with which Cummings’ views were presented and the effect this may have had on reader responses to the points being raised.

## Introduction

For reasons both historical and conceptual, genetic science is often misunderstood by the general public (Tabery 2015). There is a false but widespread belief that traits that show genetic influence are in some way pre-determined and unmalleable. This misconception can generate hostility and a fear that opening the door to genetic research will open a door to discrimination (Tabery 2015; Munafo 2016). When this controversial topic is combined with education, something most people have personal experience of and opinions about, then this is highly likely to evoke conflicting views and attitudes. At a time when scientists are calling for more account to be taken of genetic research in education (Asbury and Plomin 2013; Thomas et al. 2015) we have only a limited understanding of how the general public feels about this and how they might react to genetically informed educational policies or practices, should they become a realistic proposition.

On 11th October 2013 an article was published in the UK *Guardian* with the headline *‘Genetics outweighs teaching, Gove advisor tells his boss’*. The article reported a leaked essay written by special advisor Dominic Cummings to the then UK Secretary of State for Education, Michael Gove. It reported Cummings’ views on the influence of genes on academic achievement and his view of teacher quality in the UK. It also focused on his opinion that government initiatives such as Sure Start had failed and that UK Higher Education lacked credibility (Wintour 2013).

The article was based on a 237 page essay written by Cummings entitled ‘Some thoughts on education and political priorities’. It is notable, for our purposes, that only five of the 237 pages of this wide-ranging document discussed genes. This was not, primarily, an essay about genetics. In the short section on genetic influence Cummings’ main argument was that the science of genetics is largely ignored in education. Few could deny that this was the case at the time of writing. He briefly discussed historic misconceptions surrounding genetics and presented findings from recent studies. His short account focused primarily on the research of UK-based behavioural geneticist, Professor Robert Plomin, and argued that the single largest factor influencing the performance of children is their genes. Cummings presented heritability estimates from Plomin’s Twin’s Early Development Study (TEDS) citing a heritability estimate of 70 % for phonics at ages 7 and 12 (Harlaar et al. 2014); 60–70 % for reading and mathematics ability at ages 7, 9 and 12 (Kovas et al. 2013); and 60 % for English, Maths and Science GCSE achievement (Shakeshaft et al. 2013) . Cummings also reported the finding that educational achievement is more heritable than IQ (Kovas et al. 2013). However, he failed to cite the relevant sources in his essay, referring to this body of work generically as Plomin’s. That said, the heritability estimates presented genuinely reflected the research, although for GCSE achievement heritability estimates were between 50 and 60 %, depending on the subject.

Cummings went beyond heritability estimates, speculating that good teachers can improve reading standards for all but that they cannot narrow the gap between individuals. This assertion is likely to have been based on the finding that individual differences in reading are primarily explained by genetic differences between individuals. Therefore, an intervention targeted at a whole country can improve average achievement but is unlikely to narrow the gap between the highest and lowest achievers as it is not in fact designed to address the gap. In this sense Cummings’ speculation was reasonable and rooted in evidence. He concluded his essay by discussing what should happen in the future when genome wide association studies (GWAS) identify particular genes associated with general cognitive ability (g), something that is beginning to happen now but is still very much in its infancy (Davies et al. 2016). He argued that this development should lead to ‘truly personalised education’ (Cummings 2013, p.73). In sum, Cummings’ essay offered a short description of some existing behavioural genetic evidence and some personal speculation based on that evidence. The information he presented was, on the whole, factually correct but its portrayal in the *Guardian* article arguably introduced some distortion.

That said, a case could be made that in his brief discussion Cummings himself painted a rather narrow picture of behavioural genetic findings relevant to learning and education that was somewhat lacking in nuance. For example, his account could be interpreted as meaning that the etiology of heritability is simple, stable and not open to influence by teachers or parents. This is not the case. Heritability estimates are not fixed because they are dependent on the environment in which children learn. If children grow up in a society in which not everybody has the right to go to school, then heritability is likely to be low and school attendance will explain a lot of variance. In a society in which all children have precisely the same experiences and opportunities then heritability will be 100 % as genes will be all that differentiates between them. Heritability does not imply a lack of malleability and teachers and parents are likely to be influential via genotype-environment correlations as well as more directly, as features of a child’s learning environment (Plomin et al. 2016). This message was not apparent in either the article or the essay on which it was based. To illustrate this point, recent research shows that the genetic contribution to GCSE achievement can be explained by factors including general cognitive ability, self-efficacy, behaviour problems, personality, well-being and perceptions of the school environment (King’s College London 2014; Krapohl et al. 2014). Furthermore, a meta-analysis of all findings from behavioural genetics, i.e. not just for educationally-relevant traits, also suggests a complex picture with heritability, shared environment and non-shared environmental factors all playing an important role in explaining human behaviour (Haworth et al. 2011; Branigan et al. 2013; Polderman et al. 2015). On average, in Polderman et al. (2015) meta-analysis, genes appear to explain roughly half of the variance in behaviour, and environment (including measurement error) the other half.

The *Guardian* article reported Cummings as saying that achievement was ‘mainly based on genetics’. This does reflect the heritability estimates presented in the essay, and the research they came from. However, this description and the statement that ‘genetics outweighs teaching’ implied a determinism that the evidence does not support and that Cummings did not claim. In the research cited genes explained somewhat more variance than environmental factors in most cases. The effects of all relevant genes combined do indeed appear to outweigh the effects of any single environmental factor (e.g. teachers). However, this does not mean that achievement is genetically determined, or that teachers don’t matter, both of which the article implied: ‘Cummings maintains that individual child performance is mainly based on genetics and a child’s IQ rather than the quality of teaching’ (Wintour 2013). Cummings did not in fact say this and the research does not support it other than to the extent that it is unreasonable to pit the effects of a single environmental factor (i.e. teaching) against *all* genetic effects (i.e. not a single genetic variant).

The article was widely discussed (Jones 2013; Toynbee 2013) and generated 3008 on-line reader comments from the public. Compared to other education articles published in the *Guardian* at the same time this article received a particularly large response. For example, an article published 8 days later, detailing an attack by the Liberal Democrats on Michael Gove’s new school reforms received *n* = 1161 comments (Helm 2013).

To date, only a small number of studies have explored public perceptions of genetics in general, and even fewer have looked specifically at public perceptions of the role of genetics in education. On the whole, existing research suggests that both the general public, and education professionals, lack comprehensive knowledge about genetics, although educational professionals have so far been relatively accurate when asked to what extent they believe nature and nurture influence learning behaviour (Condit 2001, 2010; Walker and Plomin 2005; Etchegary et al. 2013).

More is known about public perceptions of genetics applied to healthcare than applied to education. Etchegary et al. (2013), looked at public opinions about genetics in a healthcare context in Canada, and found that participants felt they did not know enough but were open to the potential value of genetic information in their own healthcare. In this study participants demonstrated a positive view of advances in genetic science (Etchegary et al. 2013). In a review of the literature on public perceptions of genetics Condit (2001) found that the public tends to approach advances in genetics with ‘cautious optimism’. This review suggested that although the public does not act with ignorance towards advances in genetics, there remains a lack of means by which new information can be incorporated into public knowledge. It is crucially important therefore that any attempt to engage the public in debates such as whether genetic information is relevant to education acknowledges and responds directly to this challenge. This is an important science communication issue. Patchy or inconsistent information leads, according to Condit (2001) to a real mix within a society with the public seeing both “promise and risk” in genetics (Critchley et al. 2014). This conclusion is particularly pertinent to the current study as the media article to which commenters were responding presented a very strong view that could easily generate misconceptions among readers. This is coupled with the fact that the article did not present Cummings’ essay wholly accurately. For example, despite the statement making up the title of the article, Cummings did not actually state that ‘genetics outweighs teaching’, only that we currently undervalue and ignore the significant role that genes have to play in influencing school outcomes (Cummings 2013).

The most comprehensive study to have been undertaken of public perceptions and opinions about genetics in the UK was carried out by the Human Genetics Commission in 2001. This is now somewhat outdated but findings can nonetheless provide us with a basic foundational knowledge of what the UK public thinks (or thought). The Commission found, using a sample of >1000 UK citizens, that the vast majority of people agreed that genetic technology can be used to diagnose and cure disease. The vast majority also supported the use of genetic technology in solving crimes and finding perpetrators.

Those with better knowledge of genetics were more likely to be supportive of the role of genetics and the use of genetic technology in a medical role. Most relevant to the current study is the finding that, when asked whether they thought nature or nurture determined intelligence, the general public tended to see a 50/50 split in influence (Human Genetics Commission 2001). In light of findings from behavioral genetics, this appears to be a relatively accurate perception (Plomin and Deary 2014; Polderman et al. 2015). It is interesting to note, on the basis of the Human Genetics Commission’s 2001 survey, that the general public appeared to accept roles for both nature and nurture, and to support the use of genetic technology. Progress in genetics has been fast since the completion of the Human Genome Project and little is known about how the public feel about genetics in the light of more recent developments.

Looking specifically at perceptions of genetics in education, Walker and Plomin (2005) explored teachers’ and parents’ perceptions of how genes and the environment influence educationally relevant behaviour. They surveyed 556 UK primary school teachers and 1340 parents and asked for their views on how genes and environment influence personality, intelligence, behaviour problems, learning difficulties and mental illness. Results showed that teachers tended to take the middle ground. They tended to place roughly equal importance on genes and environment, except in the case of behaviour problems where more emphasis was placed on environmental influences. Results for parents were very similar to those for teachers (Walker and Plomin 2005). Teacher and parent views are largely in line with findings from twin studies and findings from behavioural genetic studies so far (Plomin et al. 2013). This suggests that there is in fact little conflict between what teachers and parents believe about the etiology of learning abilities and what the research shows. This does not, however, lead to a lack of controversy particularly when the message is framed in a controversial or adversarial way as it was in this *Guardian* article. When strong reactions are likely to emerge it is worth thinking about their origins. It has been argued that the eugenics movement generated distrust towards genetic science in society (Critchley et al. 2014; Tabery 2015) causing the public to shy away from anything that represents a deterministic view of genetic influence. It is important to remember this in the context of the article on which the current study is based, which at least implies genetic determinism. However, commentators also stress the fact that much of the distrust surrounding genes and genetic science stems from a lack of understanding among the public (Asbury and Plomin 2013; Asbury 2015; Tabery 2015; Ritchie 2015). Mixed levels of understanding and misunderstanding are very likely to provoke a particularly high level of debate and disagreement.

The current study was designed to explore reactions to Cummings’ views as presented in the *Guardian* article, regardless of whether his views were represented accurately, with a view to shining a light on the attitudes and beliefs of a section of UK society more than 15 years on from the Human Genetics Commission (2001) report, and more than a decade on from Walker and Plomin (2005) study of teacher and parent beliefs. It is clear from the number of reader comments that Cummings’ opinions sparked a great deal of debate among members of the public. The strongly worded and somewhat inflammatory reporting of his comments make it an ideal case for an exploratory study of public attitudes to consideration of genetics in education. It is worth noting, in considering readers’ comments, that it was clear that only a small number of commenters had actually read the original thesis by Dominic Cummings. There is also little evidence that many were familiar with the studies on which his comments were based.

### Research questions and hypotheses

The current study was designed to address three main research questions:

1. To what extent did readers’ comments support or oppose Cummings’ reported opinions?
2. To what extent was there disagreement/agreement among the readers’ comments?
3. Were objections to Cummings’ reported comments about genes more common than objections to his reported views about teacher quality, quality of higher education and wasted government initiatives?

Hypotheses were as follows:

1. The majority of commenters will disagree with Cummings’ reported comments on all educational topics covered in the article.
  This hypothesis is based on the fact that the newspaper (The *Guardian*) is politically left-leaning and the advisor is associated with a right-wing political party (The Conservatives). Moreover, the reporting of the antagonistic nature of the advisors’ comments was likely to prompt comments from those with strong adversarial reactions who wished to air their views.
2. Most commenters will disagree with Cummings’ comments on genetics in particular.
  This hypothesis is based on literature detailing the somewhat controversial nature of discussing the relationship between genes and school outcomes (e.g. Tabery 2015). It is also based on the fact that twin study research paints a much more complex picture of the topic than is evident from the article.
3. There will be evidence of much debate and disagreement between commenters across all topics (genetics, teacher quality, weakness of higher education and usefulness of government initiatives).
  This is likely because of the diversity of the sample (the opportunity to comment was open to all members of the general public) and the range of different perspectives, knowledge bases and viewpoints likely to be represented.

Overall, the aim of the current study is to provide new insight into spontaneous public reactions to the idea of embracing genetics as a relevant consideration in the planning and delivery of education. At a time when genetic research into factors such as intelligence and educational attainment is gathering pace, at both the behavioural and the molecular level, (Benyamin et al. 2013; Kirkpatrick et al. 2014; Knapton 2015; Okbay et al. 2016) it is important to understand how the public feels as a foundation for planning society’s response to these scientific developments. It is also important to try to understand whether the accuracy and tone of the reporting affects readers’ responses as this has important implications for science communication.

## Methods

A content analysis of readers’ comments underneath the online article was conducted. Content analysis is the method most widely and successfully used for analysing documents and online content (Bryman 2012; Liamputtong 2013) and has the benefit of providing a basis for future longitudinal analysis. Should a similar article emerge in the future it could be easily analysed using a very similar coding scheme and the results directly compared. The opportunity to assess whether attitudes change over time would be valuable.

A systematic sampling technique was used in which the first 100 comments from every 3rd page as listed below the article in the comments section (comments pages 1, 4, 7, 10 and 13) were copied and pasted into a chart with each comment being assigned an identification number. It was subsequently decided to bolster the sample through the inclusion of a further 75 comments from each of pages 2, 5, 8 and 11 in order to create a final sample of *n* = 800 comments. Inclusion of the 300 comments on pages 2, 5, 8 and 11 meant that results could be accepted with a 99 % confidence interval of 4.0. A confidence interval of 4.0 implies, for example, that if 50 % of commenters said they disagreed with Cummings the researchers could be 99 % certain that in the true population 46–54 % of people would disagree with Cummings.

Prior to analysis a coding framework was developed. The decision to include particular codes was driven by the primary purpose of the study–to take the opportunity to gain an insight into the general public’s perceptions of genetics in education. A deductive approach was therefore taken in line with this specific focus.

In order to be able to compare the amount of discussion of genetics with other topics also raised in the article it was necessary to include codes relating to all of the main themes mentioned (government initiatives, higher education and teaching quality). In order to be able to gauge the amount of discussion and debate that occurred between participants, to help gauge the relative level of controversy surrounding genetics, codes for comments between participants on relevant topics were also necessary. A final group of organisational codes was created to help the research team to gain a sense of comments that were not directly relevant to the research aims.

After a small amount of alteration and the addition of two new codes (‘User makes a comment that makes reference to Nazi Germany, Hitler or WW2’ and ‘Comment that corrects factual or grammatical point but expresses no individual opinion’) that emerged after coding began, the final list of codes was established and is shown in Table 1.

**Table 1:** Full list of codes identified within the readers’ comments

Code number	Code title
Comments directly related to article and the advisor’s comments:	
2	Comments opposing Cummings in the article overall
3	Comments supporting Cummings in the article overall
4	Comments opposing advisor’s comments on genetics
5	Comments supporting advisor’s comments on genetics.
6	Comments opposing advisor’s comments on teaching quality.
7	Comments supporting advisor’s comments on teaching quality.
8	Comments opposing advisor’s comments on quality of higher education.
9	Comments supporting advisor’s comments on quality of higher education.
10	Comments opposing advisor’s comments on government initiatives.
11	Comments supporting advisor’s comments on government initiatives.
Comments between participants (threads) on the comments section:	
12	Comments opposing another user who supports the advisor’s comments.
13	Comments opposing another user who opposes the advisor’s comments.
14	Comments supporting another user who supports the advisor’s comments.
15	Comments supporting another user who opposed the advisor’s comments.
16	Comments supporting a comment that promotes advisor’s genetics view.
17	Comments supporting a comments that opposes advisor’s genetics view.
18	Comments opposing a comment that promotes advisor’s genetics view.
19	Comments opposing a comments that opposes advisor’s genetics view.
Additional codes	
20	User makes criticism of government in general (beyond just Education)
21	User makes a comment that makes any reference to Nazi Germany, Hitler or WW2.
22	User makes a troll (In internet slang, a troll is a person who promoted disagreement on the internet by starting arguments or upsetting people. They may also post inflammatory or irrelevant messages) comment or any form of personal attack (looks, background, ethnicity, sexual orientation .etc. This includes comments about the appearance of Cummings or government members)
23	User makes a completely unrelated comment to the article content or another user’s comments.
24	Comment that corrects factual or grammatical point but expresses no individual opinion.

Once the coding framework was established a single researcher carefully read and coded each individual reader comment. Every comment was also assigned a code showing whether it was an original comment directed at the article or a thread (i.e. a comment directed at another user’s comment). On the whole, comments tended to fit into at least two coding categories.

Once the first researcher had assigned codes to all of the 800 comments sampled, it was necessary to check that coding was reliable. A second researcher coded a proportion of the data for the purpose of checking inter-rater reliability. This researcher read the original source (the online news article) and discussed it with the first researcher in order to become familiar with the context. The coding framework was then introduced and the researcher was asked to focus on coding comments for the nine most salient codes, those relating directly to the research questions (i.e. not organisational codes). Ten percent of the sample (80 comments) were coded independently in this way and both coders’ decisions were entered into SPSS in order to assess inter-rater reliability.

During the data entry process a very small number of edits (*n* = 8) to the original coding were made, based on differences between coders. This reconciliation and reflection stage (Hruschka et al. 2004) allowed the original coder the opportunity to reassess a small number of original judgements in light of disagreement, and to make adjustments where appropriate. This resulted in an overall percentage change in the final results for only one code (21-User makes a comment that makes any reference to Nazi Germany, Hitler or WW2). Cohen’s Kappa was used as the statistical test of inter-rater reliability. Each of the nine codes was analysed individually to assess the Kappa score. On the whole, based on parameters established in the literature, results suggested agreement was ‘good’ or ‘very good’ (McHugh 2012). However, one test suggested only ‘fair’ agreement and another was not possible to conduct as there was not enough variance in the codes (the code was judged as not being present in almost all of the sample). However, overall there was an acceptable level of inter-rater reliability (Table 2).

**Table 2:** Kappa figures for codes used for the inter-rater reliability process

Inter-rater code	Measure of agreement–Kappa
Comments opposing Cummings in the article	0.26
Comments supporting Cummings in the article	1
Comments opposing advisor’s comments on genetics	0.735
Comments supporting advisor’s comments on genetics	0.627
Comments supporting a comment that promotes the advisor’s genetics view	No codes present in sample
Comments supporting a comment that opposes advisor’s genetics view	0.64
Comments opposing a comment that promotes advisor’s genetics view	0.794
Comments opposing a comment that opposes advisor’s genetics view	0.82
Reader makes Nazi reference	0.874

Table 3 provides an example of some of the more frequently occurring codes and how they were defined. A number of typical examples have been shown to illustrate the nature of the comments that were sorted into the different coding categories.

**Table 3:** Example of deductive codes and operational definitions

Code	Operational Definition	Example
Comments opposing the article overall	The overall tone of the comment suggests general disagreement with the advisor–may not be specific	‘Clearly this man has no idea what he is talking about. There is no evidence whatsoever. Quite frankly he is full of crisp…’
Comments supporting the article overall	The overall tone of the comment suggests general agreement with the advisor–may not be specific.	‘The first few pages are not drivel [referring to the original thesis]. They discuss the importance and difficulty of managing complex organisations, and the idea of Odyssean education is interesting, at the very least.’
Comments opposing advisor’s comments on genetics	Makes direct reference to the genetic content of the article and expresses a disagreement towards it.	‘The idea that genetics plays a role in intelligence, is a dangerous ideology that can be used to afford false weight to elitist class discrimination, and ideas of superiority of certain genetically related groups…’
Comments opposing a comment that promotes advisor’s genetics view	Thread–one commenter disagrees with another commenter who has displayed support for the advisor’s views on genetics.	“’Genetics plays a role in intelligence”–‘’An inconvenient truth for Guardian readers”. Why? I don’t see this at all. The ‘left’ want fair chances for all–dim or geniuses…’
User makes criticism of government in general	Shows a general disagreement towards the Coalition government beyond the advisor’s reported opinions.	‘They’re heading towards an extremely dark place at an incredible pace and they’re dragging this country along for the ride.’
User makes a comments that makes any reference to Nazi Germany.	Makes any reference, positive or negative, that is related to the Nazi genetics movement.	Genetics doesn’t play a Role, it’s a myth that Black athletes are better at some sports than Whites, we’re all EXACTLY equal, unt any von who diasagrees vis me ist eine racist and should be put in ze koncentrstion camp unt re educated.’

Once all 800 comments had been assigned a code/codes then descriptive univariate analyses were conducted to provide a clear picture of the distribution of opinions/attitudes and the overall frequency of particularly strong views such as Nazi-related comments. Overall, analysis focused on the nature of original threads, and on the amount of agreement/disagreement within and between reader comments.

## Results

Within the sample there was an almost equal split between comments that were original comments directed at the article (52 %) and comments that were threads (48 %), that is, comments directed at another comment. It was found that 34 % of reader comments in the sample contained unrelated information (troll, personal attack or unrelated) or were grammar/fact corrections that expressed no relevant opinion. It is important to bear this in mind as it is the remaining 66 % of reader comments (*n* = 528) that are of real interest and relevance to the current study.

**Fig. 1:**
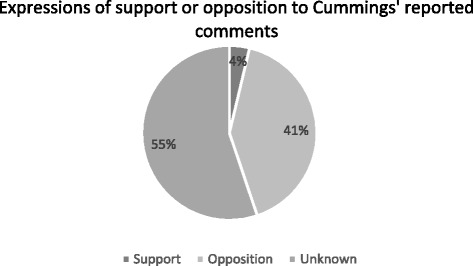
Overall distribution of support of opposition to Cummings’ reported comments

Looking at comments made generally by readers about the article, that is, not linked to one of the specific topics analysed–frequency analysis showed that 41 % of reader comments expressed clear opposition to Cummings’ general position as portrayed in the article, and only 4 % showed clear support for his overall position as portrayed in the article. The remaining 55 % of sampled commenters did not express anything that would allow a conclusion to be drawn as to whether they supported or opposed the overall tone and content of the article (see Fig. 1).

**Fig. 2:**
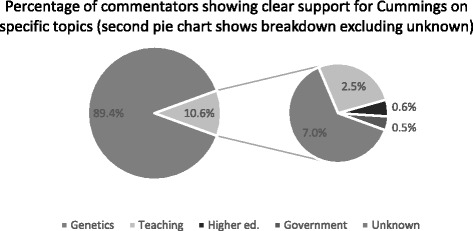
Distribution of support towards Cummings on the specific topics presented in the article–percentages in pie of pie show proportion of overall comments that showed clear support for specific topics

Looking specifically at the amount of support/opposition for Cummings’ views on the individual topics covered by the article (genetics, teaching quality, standards of higher education and government initiatives), results showed a clear focus on genetics as the ‘hottest’ topic of discussion among reader comments. Overall, however, 89.4 % of sampled comments (*n* = 715) did not clearly express support for Cumming’s reported view on any topic. This left 10.6 % (*n* = 85 comments) which expressed specific support for his view, as presented in the article, on at least one topic. We see that genetics received the most comments with 7 % (*n* = 56) of the overall sample specifically supporting Cummings’ reported views. His views on teaching quality generated 20 comments (2.5 % of sample) showing support for his position. His view on government initiatives generated only four supportive comments (0.5 % of sample) and on higher education just 5 (0.6 % of sample). It is worth noting that overall support for the general content and tone of the article and specific support for a particular topic were coded separately. It was possible for a commenter to agree with Cummings on an individual topic but to express overall disagreement with his position, and vice versa (Fig. 2).

**Fig. 3:**
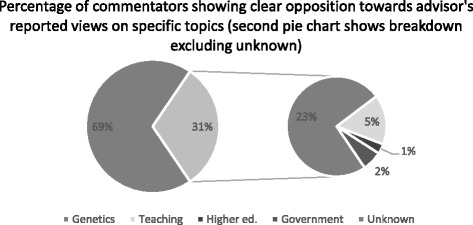
Distribution of opposition towards Cummings on the specific topics presented in the article–percentages in pie of pie show proportion of overall comments that showed clear opposition for specific topics

The proportion of those agreeing with Cummings’ reported views on genetics was double that of the second most supported topic–teaching quality. A similar pattern was seen in comments which expressed clear opposition to Cummings’ reported view on one of these topics. Percentage breakdowns regarding expressed opposition to Cummings’ reported views are displayed in Fig. 3. Overall 69 % (*n* = 552 comments) did not clearly express opposition to a particular topic mentioned in the article. This left 31 % (*n* = 248 comments) which specifically opposed one or more of Cummings’ specific views. Once again we see that genetics received the most comments with 23 % of the sample (*n* = 184 comments) specifically opposing his reported views on genetics. Teaching quality received 5 % (*n* = 40) of overall comments showing clear opposition towards Cummings’ reported views, government initiative opposition only 2 % (*n* = 16) and higher education just 1 % (*n* = 8). It is clear that Cummings’ reported views generated more opposition than they did support.

The next step was to look at the amount of agreement and disagreement between reader comments, that is, in threads in which readers responded to each others’ views rather than directly to the article. For this purpose, two codes for agreement and two codes for disagreement were combined. Commenters that either agreed with other commenters who expressed support for Cummings’ views, or disagreed with commenters expressing disagreement, were combined and coded as being ‘in agreement’ with Cummings. Conversely, commenters that either agreed with others who expressed disagreement with Cummings, or disagreed with supportive comments, were coded as ‘disagreeing’ with Cummings’ view. On this basis, 10 % of threads suggested overall opposition to the article and 3.3 % suggested overall support (Table 4).

**Table 4:** Demonstration of support/opposition towards the advisor’s comments on genetics through agreement/disagreement between user in threads

Code	Present (%)	Not Present (%)	Overall support (%)
Comments supporting a comment that promotes advisor’s genetics view	0.3	99.7	3.3
Comments opposing a comments that opposes advisor’s genetics view	3	97	
			Overall opposition (%)
Comments supporting a comment that opposes advisor’s genetics view	5	95	10
Comments opposing a comment that promotes advisor’s genetics view	5	95	

An example of a thread in which a commenter disagreed with a previous commenter who, in turn, had disagreed with Cummings’ reported view is shown here for illustrative purposes:

***Original comment:*** *‘Ridiculous, genetics may be a starting point but anyone (except those with brain damage/seriously disabled) can become as good as someone else if they have the proper opportunities and try hard.’*

***Response on thread:*** *‘Think about the people you have met over the years. Is this really true?’*

Finally, it is worth noting that 6.8 % of reader comments (*n* = 54) made a clear reference to Nazis or eugenics in their response to the article. On the whole these references suggested that Cummings’ reported views about genetics in education were comparable to Nazi ideology. For example:

*‘In the 30’s they called this Eugenics, and it was part of the nazi education. Oooops’*

*‘Yes it seems to be verging more than a little into ‘master race’ territory.’*

A number of these references appeared to be made sarcastically, but these too insinuated the same concern. For example:

*‘Aaah. The joy of facism, They’ll be doing experiments on the untermensch [German term for someone considered racially or socially inferior] soon....As if they’re not already’.*

A tiny handful of references to Nazis however, did explicitly challenge the comparison of Cummings to Nazis/Hitler. For instance:

*‘Genetics plays a role in intelligence. How on earth can anyone deny that this is the case? Accepting this as scientifically probable doesn’t mean you are a member of the Nazi Party. An uncomfortable truth but a truth nonetheless.’*

On the whole, of the 54 reader comments making an explicit reference to Nazis Hitler, WW2 or eugenics (6.8 % of full sample) almost all did so as a criticism of Cummings, or an expression of fear. The shadow of eugenics remains clear.

## Discussion

The results of this content analysis clearly indicate that even when included as just one of a range of potentially controversial educational topics, genetics evokes a disproportionate amount of debate, as argued by others (Asbury and Plomin 2013; Tabery 2015). This supports the study’s third hypothesis that there would be a great deal of debate and disagreement among reader comments about Cummings’ opinions as presented in the article. Both our first and second hypotheses, that most people would disagree with Cummings and that his comments regarding genetics would generate a particularly high amount of disagreement, were also supported. Genetics did prove to be the topic that most people specifically disagreed with. However, it is interesting to note that it was also the topic that Cummings received the most support on.

These findings mirror the findings of Condit (2001) who, having reviewed the literature on public perceptions of genetics, found no clear viewpoint but rather a mix of opinions. The fact that genetics was the most discussed of all topics presented in the article may be due, to some extent, to the inclusion of genetics–in a particularly provocative way–in the headline. However, in order to comment in a meaningful manner, at least a glance at the whole article would be necessary. The article itself contained a total of ten paragraphs of which only two talked specifically about Cummings’ views on genetics in education. Moreover, these two paragraphs did not appear until near the very end of the article. This shows that even when discussed in the midst of other important topics, genetics remained a key focus of discussion and debate relative to the other topics discussed.

From the limited literature on public perspectives on genetics in education, the current findings make sense. A lack of knowledge and understanding can often lead to hostility on a complex subject like genetics (Tabery 2015). Moreover, Walker and Plomin (2005) found that most teachers and parents see genes as just half of the explanation for individual differences in educationally relevant behaviour (this was also found in the Human Genetics Commission survey 2001), so when Cummings was reported as saying that genes are substantially more important than environment, disagreement was likely This disagreement was likely further inflamed by the fact such views from a right wing political advisor were presented in a left leaning newspaper. For many, the suggestion that teachers and schools may not be highly influential is a hard concept to swallow. It could suggest (mistakenly) that we have little control over our destinies and that we are unable to influence the future of society’s children even via education which is our biggest and most expensive social intervention. Over-simplified and misleading reporting of Cummings’ message is likely to have exacerbated this misconception and generated further hostility. Results from behavioural genetic studies show that genes are rarely deterministic and that heritability does not imply immutability. Nor is it ever suggested that the environment does not have a vitally important role to play in education (Asbury and Plomin 2013). Genes work through environments and it is often difficult to disentangle their effects. However, the evidence for the effects of both is undeniable. It is complexities such as these that both Cummings and The *Guardian* failed to properly make clear, and this may have been a contributory factor in some of the negative reactions the article received. A more balanced portrayal of the evidence, with an emphasis on the fact that heritability is a population statistic and does not imply a lack of malleability, may have generated a positive or open reaction from those who (with good reason) fear a link between genetic determinism and discrimination. In this article we see a prime example of over-simplification of science leading to hostility and misconceptions from the general public, particularly in a field such as education which the majority of the British public value and place great importance on. We perhaps also see the damage that can be done by having newspaper headlines written to be attention grabbing rather than to accurately represent the content of the article.

The high number of comments (and threads) generated by this article illustrates the amount of discussion and debate that this article generated. For comparison, a related article that subsequently appeared in the Guardian, also talking about the role of genetics in education with the word ‘genes’ in the headline but from a pro-nurture stance, received only *n* = 763 comments (James 2016). It is advocating the influence of nature that appears to provoke particularly high levels of debate. Many threads under the Cummings article were made up of numerous commenters going back and forth in their discussions. This suggests that genetics in education is of particular interest and importance to the general public as found in previous literature (Condit 2001; Human Genetics Commission 2001).

It was also interesting to note the occurrence of reader comments related to Nazis, Hitler and eugenics. On the whole these were used as an expression of disagreement or anger at Cummings’ comments on genetics in education. Although these reader comments demonstrate the strength of disagreement and anger felt by some readers they perhaps also suggest a misunderstanding or lack of knowledge of the difference between the suggestion that genetics plays an important role in learning abilities and achievement (as suggested by Cummings) and the misuse and abuse of genetics for harm by the Nazis. This lack of knowledge, prevalence of misunderstanding, hostility and fear around genetics was one of the key points to emerge from the literature (Condit 2001, 2010; Etchegary et al. 2013; Asbury and Plomin 2013; Asbury 2015; Tabery 2015) so it is valuable to see it demonstrated clearly in such a public arena. It is worth remembering that Cummings’ comments on the role of genetics are largely based on reliable scientific evidence (even if somewhat inflated by the reporting in this article) and are not in any sense extreme or unsubstantiated views. This kneejerk reaction in a notable minority of reader comments is likely to be based on fear and misunderstanding and suggests that geneticists may have a significant public engagement challenge that needs to be addressed if the public are to embrace their science in the context of education. However, it is also worth noting that some of the reactions within the article may have been politically rather than scientifically motivated and there remains a need for further research to establish the true nature of public understanding and misunderstanding of genetics.

### Limitations

Due to the politically partisan nature of the newspaper in which the article was published, and some emotive and misleading reporting, the generalisability of these findings are limited. It could be argued (with the advisor working for a right wing party) that such hostile reactions were to be expected. Although this must be taken into account it remains unknown what sort of reaction might be received in another newspaper. Given the divisive nature of the topic it seems likely that, even if Cummings’ comments been reported in a right wing newspaper, reactions would have been mixed. It would be interesting to test this hypothesis in future research with a relevant article.

We cannot say that the views represented in the sample are those of the UK population at large but they do represent a snapshot of how a large (self-selecting) sample of the general public reacted to a story suggesting a deterministic view of genetics in education. The inclusion of the misconstrued statement that ‘genetics outweighs teaching’ is also likely to have drawn commentators who had a specific interest in the role of genetics in education or a vested interested in teaching.

### Future research

Directions for future research could involve a comparative analysis of reader comments from related sources. For instance, analysis of a similar article in a more right-wing newspaper could provide a valuable insight into potential differences in public opinion based on political allegiance. Analysis of an article in favour of environmental determinism in a left-leaning paper could also prove interesting and the James (2016) article cited earlier could be a good candidate for this. Moreover, the method of comments analysis used is well suited to longitudinal analyses and analysis such as the one reported in this paper could easily be repeated over time in response to the emergence of new articles relating to behavioural genetics in education. This could be a useful and interesting way of documenting stability and change in public perceptions as this science advances.

Overall, findings from this study contribute to the currently limited picture of how the general public feel about the potential application of genetics in an educational context. The data suggest that, as predicted, this topic evokes particularly strong feelings and that at least some opposition may be based on misunderstanding. More commenters disagreed than agreed with the views expressed by Cummings, in spite of the empirical evidence supporting any statement that genes play a substantial role in explaining individual differences in academic achievement (</ = 50 %) and that, although environmental factors (and measurement error) explain all of the remaining variance, teaching is just one aspect of the child’s environment that also includes society, school, home and even prenatal environments (Turkheimer 2000; Kovas et al. 2007; Plomin and Deary 2014). Hostility and misunderstanding needs to be addressed effectively by researchers in behavioural genetics, particularly those who hope their research could have a positive impact on education. In this sense, it is hoped that the current study can provide some useful insight for those wishing to spread their research and findings beyond the scientific community to the general public.
